# Pre-hatch thermal manipulation of embryos and post-hatch baicalein supplementation mitigated heat stress in broiler chickens

**DOI:** 10.1186/s40104-023-00966-6

**Published:** 2024-01-22

**Authors:** Sadid Al Amaz, Ajay Chaudhary, Prem Lal Mahato, Rajesh Jha, Birendra Mishra

**Affiliations:** Department of Human Nutrition, Food and Animal Sciences, College of Tropical Agriculture and Human Resources, University of Hawai’i at Manoa, AgSci 216, 1955 East-West Rd, Honolulu, HI 96822 USA

**Keywords:** Baicalein, Growth performance, Gut microbiota, Heat stress, Thermal manipulation

## Abstract

**Background:**

High environmental temperatures induce heat stress in broiler chickens, affecting their health and production performance. Several dietary, managerial, and genetics strategies have been tested with some success in mitigating heat stress (HS) in broilers. Developing novel HS mitigation strategies for sustaining broiler production is critically needed. This study investigated the effects of pre-hatch thermal manipulation (TM) and post-hatch baicalein supplementation on growth performance and health parameters in heat-stressed broilers.

**Results:**

Six hundred fertile Cobb 500 eggs were incubated for 21 d. After candling on embryonic day (ED) 10, 238 eggs were thermally manipulated at 38.5 °C with 55% relative humidity (RH) from ED 12 to 18, then transferred to the hatcher (ED 19 to 21, standard temperature) and 236 eggs were incubated at a controlled temperature (37.5 °C) till hatch. After hatch, 180-day-old chicks from both groups were raised in 36 pens (*n* = 10 birds/pen, 6 replicates per treatment). The treatments were: 1) Control, 2) TM, 3) control heat stress (CHS), 4) thermal manipulation heat stress (TMHS), 5) control heat stress supplement (CHSS), and 6) thermal manipulation heat stress supplement (TMHSS). All birds were raised under the standard environment for 21 d, followed by chronic heat stress from d 22 to 35 (32–33 °C for 8 h) in the CHS, TMHS, CHSS, and TMHSS groups. A thermoneutral (22–24 °C) environment was maintained in the Control and TM groups. RH was constant (50% ± 5%) throughout the trial. All the data were analyzed using one-way ANOVA in R and GraphPad software at *P* < 0.05 and are presented as mean ± SEM. Heat stress significantly decreased (*P* < 0.05) the final body weight and ADG in CHS and TMHS groups compared to the other groups. Embryonic TM significantly increased (*P* < 0.05) the expression of heat shock protein-related genes (*HSP70, HSP90,* and *HSPH1*) and antioxidant-related genes (*GPX1* and *TXN*). TMHS birds showed a significant increment (*P* < 0.05) in total cecal volatile fatty acid (VFA) concentration compared to the CHS birds. The cecal microbial analysis showed significant enrichment (*P* < 0.05) in alpha and beta diversity and *Coprococcus* in the TMHSS group.

**Conclusions:**

Pre-hatch TM and post-hatch baicalein supplementation in heat-stressed birds mitigate the detrimental effects of heat stress on chickens' growth performance, upregulate favorable gene expression, increase VFA production, and promote gut health by increasing beneficial microbial communities.

**Supplementary Information:**

The online version contains supplementary material available at 10.1186/s40104-023-00966-6.

## Introduction

The continuous rise in global temperature and the abrupt shift in the climate pattern over the last century has led to a significant change in the trajectory of heat tolerance in humans and animals. Amongst the domesticated animal species, chickens are most vulnerable to heat stress (HS) due to their inadequate thermoregulatory capacity. HS makes it challenging for broiler chickens to achieve their full growth performance potential. Also, HS in poultry suppresses the natural immune response, alters the acid–base balance, and causes oxidative damage that reduces feed consumption, carcass quality, and meat quality [1, 2]. It indicates that the birds' growth performance is hindered due to their thermotolerance being overtaxed, leading to economic losses due to increased morbidity and mortality [3].

It is nearly impossible to eliminate heat stress in poultry farms even though they are raised in environmentally controlled houses. Numerous dietary, managerial, and genetic strategies have been used to mitigate the adverse effects of heat stress on chickens. Reducing heat stress using managerial and genetic strategies is expensive, time-consuming, and laborious. Therefore, an embryonic thermal manipulation (TM) strategy has been tested to increase the heat tolerance capacity in poultry. In TM, embryos are exposed to a relatively higher incubation temperature at critical stages of embryonic growth [4]. TM appears most effective during the crucial period of hypothalamus-hypophysis-thyroid or adrenal axis development or both [5]. So, TM timing must be linked to the onset of the hypothalamus-hypophysis-thyroid axis to alter the thermotolerance threshold capacity [6]. It is reported that TM birds have enhanced thermotolerance [7], improved hatchability, growth performance, muscle development, immunocompetence, and overall welfare [8].

Dietary supplementation strategy to mitigate the heat stress effects in broiler chickens is well-documented [9]. Baicalein (5,6,7-trihydroxyflavone) is a primary flavonoid derived from the root of *Scutellaria baicalensis* Georgi (Chinese skullcap or Huang Qin). Baicalein has been shown to exert multiple pharmacological effects and could be a potential feed additive for broilers. Baicalein supplementation in broiler diets enhanced their growth, immune response, serum lipid metabolism, and antioxidant profile [10] and reduced abdominal fat accumulation [11].

Therefore, based on the effectiveness of embryonic TM and post-hatch baicalein supplementation, we hypothesized that TM would alleviate the detrimental effects of heat stress by altering the hypothalamus set point and post-hatch baicalein supplement, along with pre-hatch TM, will improve the production performance. This study aimed to investigate the underlying mechanism of pre-hatch TM and post-hatch baicalein supplementation, assess the thermotolerance by measuring the relative gene expression, and evaluate the growth performance and health parameters.

## Materials and methods

### Experimental design

Fertile Cobb 500 eggs (*n* = 600) were sourced from a local hatchery (Asagi Hatchery Inc., Honolulu, HI, USA). All the eggs were randomly placed into 3 incubators and immediately incubated (GQF incubator, Savannah, GA, USA; 200 eggs each) at standard temperature (37.5 °C) and 55% relative humidity (RH) for the first 11 embryonic days (ED). On ED 10, the eggs were candled to separate the live embryos (*n* = 474) to be used in the study. On ED 12, eggs were divided into two incubation groups: 1) Control (*n* = 236) (standard temperature until the hatch day, ED 21), and 2) TM group (*n* = 238) (38.5 °C at 55% RH, 12 h/d, from ED 12 to ED 18 and standard temperature from ED 19 to ED 21) in 2 incubators for each treatment with automatic temperature control, 55% RH, and egg turning (every 2 h). The experiment was conducted using a completely randomized block design.

### Hatching and rearing management

**Fig. 1:**
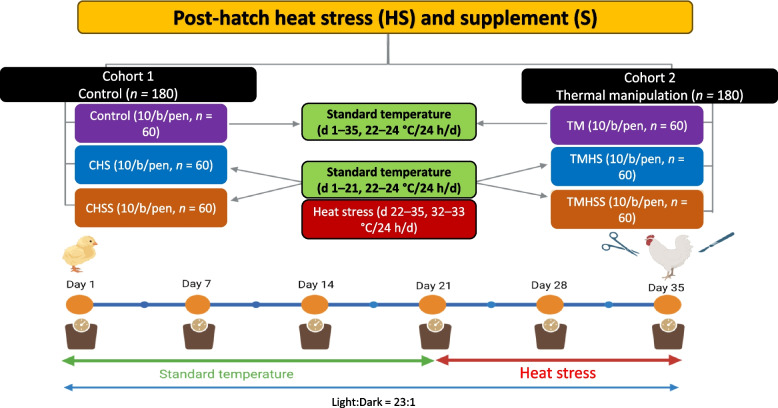
Post-hatch heat stress and supplement strategy (Created by biorender.com)

After hatch, unsexed day-old chicks (*n* = 360) were divided equally into two primary cohorts: 1) Control (*n* = 180 from Control group at hatch) and 2) TM (*n* = 180 from TM group at hatch). A total of 6 treatment groups were made, 3 groups from each Control and TM cohort at hatch. So, the post-hatch treatments were: 1) Control, 2) control heat stress (CHS), 3) control heat stress supplement (CHSS), 4) thermal manipulation (TM), 5) thermal manipulation heat stress (TMHS), and 6) thermal manipulation heat stress supplement (TMHSS). All the chicks were weighed individually, winged tagged, and randomly placed in 36 pens (10 birds per pen), making 6 replicates for each treatment group (*n* = 60 birds per treatment). Chicks were raised on the litter floor pen system following standard Cobb-500 broiler rearing and management guidelines. On d 21, birds in the CHS, CHSS, TMHS, and TMHSS groups were exposed to cyclic heat stress (33–35 °C) from 8:00 to 18:00 h (to mimic the environmental temperature) and 22–24 °C during the night with 55% RH till d 35. Control and TM groups were raised at standard room temperature (22–24 °C) with 55% RH throughout the study. Birds were monitored three times daily (morning, afternoon, and evening) to ensure proper management and health conditions. The pens were completely randomized in this study. Birds were raised with a standard lighting system (23 h light:1 h dark). The experimental design and management strategy are shown in Fig. 1.

### Diet

The corn-soybean meal-based basal diets were formulated in two phases: starter (d 1–21) and finisher (d 22–35) to meet the nutritional requirements of the Cobb 500 broilers [12]. Feed and water were supplied ad libitum throughout the study. Control, CHS, TM, and TMHS groups were fed the basal diet throughout the study. CHSS and TMHSS groups were fed baicalein supplemented (250 mg/kg) in basal diets throughout the study. The dose of the baicalein supplement was chosen based on its antioxidant properties and the dose rate used in rodents, cattle, and human studies. The diet’s composition and nutrient profile are presented in Table 1.

**Table 1:** Composition of experimental diets and their nutrient profile

Ingredients, %	Starter diet (1–21 d)		Finisher diet (22–35 d)	
	**Control**	**Test**	**Control**	**Test**
Corn	53.67	53.67	60.84	60.84
SBM	38.00	38.00	31.00	31.00
Soybean oil	5.00	5.00	5.50	5.50
Limestone	1.35	1.35	1.20	1.20
Monocalcium phosphate	0.75	0.75	0.44	0.44
Lysine	0.18	0.18	0.10	0.10
Met	0.18	0.18	0.13	0.13
Thr	0.04	0.04	0.00	0.00
Tryptophan	0.00	0.00	0.00	0.00
Choline Cl	0.00	0.00	0.00	0.00
NaCl	0.20	0.20	0.18	0.18
Sodium bicarbonate	0.12	0.12	0.10	0.10
Vitamin and mineral premix^1^	0.50	0.50	0.50	0.50
Baicalein	0.00	0.00020	0.00	0.00020
Phytase	0.01	0.01	0.01	0.01
Total	100.00	100.00	100.00	100.00
Nutrients contents in the diet, %				
AMEn, kcal/kg	3,040	3,040	3,165	3,165
CP	21.47	21.47	18.54	18.54
Ca	0.91	0.91	0.77	0.77
Total P	0.71	0.71	0.61	0.61
Available P	0.45	0.45	0.37	0.37
Lys	1.32	1.32	1.09	1.09
Met	0.52	0.52	0.44	0.44
Cys	0.42	0.42	0.40	0.40
Thr	0.87	0.87	0.73	0.73
Trp	0.31	0.31	0.27	0.27
Met + Cys	0.92	0.92	0.82	0.82
Arg	1.55	1.55	1.35	1.35
Val	1.18	1.18	1.05	1.05
Ile	0.90	0.90	0.78	0.78
Leu	1.82	1.82	1.66	1.66
NDF	8.86	8.86	8.73	8.73
CF	3.84	3.84	3.51	3.51
Na	0.16	0.16	0.14	0.14
Cl	0.16	0.16	0.15	0.15
Choline, mg/kg	1,371	1,371	1,224	1,224
Digestible Lys, %	1.17	1.17	0.95	0.95
Digestible Met, %	0.48	0.48	0.40	0.40
Digestible Thr, %	0.67	0.67	0.55	0.55

^1^Provides following nutrients (per kg of diet): vitamin A (*trans*-retinyl acetate), 10,000 IU; vitamin D_3_ (cholecalciferol), 3,000 IU; vitamin E (all-*rac*-tocopherol-acetate), 30 mg; vitamin B_1_, 2 mg; vitamin B_2_, 8 mg; vitamin B_6_, 4 mg; vitamin B_12_ (cyanocobalamin), 0.025 mg; vitamin K_3_ (bisulfate menadione complex), 3 mg; choline (choline chloride), 250 mg; nicotinic acid, 60 mg; pantothenic acid (D-calcium pantothenate), 15 mg; folic acid, 1.5 mg; betaine anhydrous, 80 mg; D-biotin, 0.15 mg; zinc (ZnO), 80 mg; manganese (MnO), 70 mg iron (FeCO_3_), 60 mg; copper (CuSO_4_·5H_2_O), 8 mg; iodine (KI), 2 mg; selenium (Na_2_SeO_3_), 0.2 mg

### Growth performance

All the birds were individually weighed on d 1 and placed in their replicate pens. Per replicate pen, body weight and feed intake were recorded weekly (on d 7, 14, 21, 28, and 35). Average daily gain (ADG), average daily feed intake (ADFI), and feed conversion ratio (FCR) during the starter phase (1–3 weeks), finisher phase (3–5 weeks), and overall period (d 1–35) were calculated based on recorded body weight and feed intake.

### Sample collection

At the end of the animal experiment (d 35), one bird from each pen (6 per treatment) was euthanized using carbon dioxide asphyxiation for sampling. Small pieces of the ileum (5 cm near the ileocecal junction) were collected, snap-frozen, and stored at −80 °C until RNA extraction. For microbial profiling and volatile fatty acid (VFA) analysis (*n* = 6 per treatment; one bird from each treatment), the cecum was isolated, wrapped separately in aluminum foil, and snapped frozen at −80 °C.

### Quantitative real-time PCR (qPCR)

According to the manufacturer's instructions, TRIzol reagent (Invitrogen, Carlsbad, CA, USA) was used to isolate total RNAs from frozen tissues (50–100 mg). Total RNA concentration was determined using NanoDrop One (Thermo Fisher Scientific, Madison, WI, USA). RNA quality was determined by running samples on 2% agarose. The RNA samples were stored at −80 °C for further analysis. The expressions of candidate genes were analyzed using qPCR (Quant Studio 3), as described previously [13]. NCBI Primer-Blast tool was used to design specific primer pairs for detecting each gene. 1 μg of total RNA (20 μL reaction of RT mixture) was reverse-transcribed into complementary DNA (cDNA) using a High-Capacity cDNA Reverse Transcription Kit (Applied Biosystems, Foster City, CA, USA) and then diluted with nuclease-free water (1:25). Using real-time PCR system (Applied Biosystems), qPCR was performed with PowerUp SYBR Green Master Mix (Applied Biosystems, Foster City, CA, USA). The qPCR reaction mixture contained 3 μL of cDNA, 5 μL of PowerUp SYBR Green Master Mix, and 1 μL of each forward and reversed primer at a concentration of 5 μmol to yield a final reaction volume of 10 μL. Standard cycling mode was utilized for the qPCR reaction. A melting curve was constructed to validate the SYBR Green-based objective amplicon. In addition, the specificity of each primer pair was determined by running the qPCR products through 1% gel electrophoresis. Three housekeeping genes: glyceraldehyde 3-phosphate dehydrogenase (*GAPDH*), beta-actin (β-actin), and TATA-box binding protein (*TBP*), were analyzed in triplicate. The most stable housekeeping gene in the ileum was TBP, which was used to normalize target gene expression. The genes of interest were analyzed in duplicate, and the average value of each experimental replicate was calculated. The expression levels of target genes were determined using cycle threshold (Ct) values normalized with TBP. The fold change of each gene was determined using the 2^−ΔΔCt^ method. The gene primers list is presented in Additional file 1.

### Volatile fatty acids

The VFA was analyzed as previously described [14]. Briefly, 200 mg of cecal content was weighed in the Eppendorf tube. Then the final volume of 1,500 μL was created by adding deionized water (1,100 μL), trimethyl acetic acid (100 μL), and metaphosphoric acid (100 μL). Following homogenization with a vortex, materials were centrifuged for 10 min at 15,000 r/min at 4 °C. A Gas Chromatography system (TRACE 1300; Thermo Scientific, Waltham, MA, USA) and supernatants (1,000 μL) were used to analyze VFA in the samples. Helium was used as a carrier gas at a 15.5 mL/min rate. Each sample's run duration was 17.5 min, with an injection volume of 0.5 μL. The initial temperature regimen was 120 °C for 4 min, followed by a 4 °C/min increase to 160 °C. 0.1, 0.5, 1, 2, 4, 6, 8, 10, 12, and 14 mmol/L of the standard stock solution mixture containing formic, acetic, propionic, isobutyric, butyric, isovaleric, valeric, isocaproic, hexanoic, and n-caproic acids were used. The software Chromeleon™ 7.2 (Thermo Scientific, Waltham, MA, USA) was used for data management and processing.

### DNA extraction and 16S rRNA gene sequencing

The DNA was extracted, and the 16S rRNA gene was sequenced as previously described [13]. Following the manufacturer's instructions, total genomic DNA was extracted from the cecal digesta using the QIAamp® DNA Stool Mini Kit (Qiagen, Hilden, Germany), as previously described [15]. NanoDrop One (Thermo Fisher Scientific, Madison, WI, USA) was used to assess bacterial DNA concentration and viability. The V3-V4 hypervariable regions of the 16S rRNA gene were amplified following the Illumina 16S Metagenomic Sequencing Library protocol (Illumina) with the following change. The PCR reaction was performed with Platinum Taq DNA Polymerase High Fidelity (Invitrogen, Life Technologies Corporation, Grand Island, NY, USA), Mag-Bind Total Pure NGS beads (Omega Bio-Tek, Norcross, GA, USA) were used for PCR clean-ups, and 35 cycles were used in PCR. Finally, the Illumina MIseq sequencer was used to sequence normalized, aggregated, and sequenced amplicons.

### DNA sequence analysis

CLC Genomics Workbench 12.0.1 and the CLC Microbial Genomics module were utilized for bioinformatics analysis of microorganisms. The procedures for sequencing analysis were carried out as outlined in the operational taxonomical units (OTUs) clustering step-by-step tutorial (Qiagen, Hildesheim, Germany). In short, the demultiplexed sequences were uploaded as fastq files into the CLC workbench, where they were paired, trimmed, and filtered to eliminate lower coverage reads. Using the CLC Microbial Genomics module, the filtered reads were grouped as OTUs based on 97% sequence similarity against the Greengenes v13_8 97% database. For alpha and beta diversity analysis, the phylogenetic tree was constructed utilizing a maximum-likelihood method based on multiple sequence alignment (MSA) of the OTU sequences generated by MUSCLE in the workbench. The alpha diversity was estimated using Simpson's index and Shanon entropy and was represented using a boxplot. Beta diversity was estimated by calculating unweighted and weighted UniFrac distances and displaying the results using principal coordinate analysis (PCA). To determine the significance of beta diversity, a permutational multivariate analysis of variance (PERMANOVA) was conducted. Differentially abundant taxa (order, family, and genus) were determined using one-way ANOVA on the OTU table after eliminating OTUs with an abundance of less than 10, and mean separation between treatment groups was performed using Fisher's Least Significant Difference test in R-studio.

### Statistical analyses

The growth performance, gene expression, and VFA data were analyzed using GraphPad (GraphPad Software, San Diego, CA, USA) and RStudio (R version 4.2.2, RStudio PBC, Boston, MA, USA). All data are presented as mean ± SEM. Following a one-way analysis of variance (ANOVA), the Tukey-HSD test was utilized to compare the means of the various treatment groups. In the CLC Microbial Genomics module, the Kruskal–Wallis pairwise test for alpha diversity and the PERMANOVA test for beta diversity were utilized. The statistical significance threshold was set at *P* < 0.05.

## Result

### Growth performance

**Fig. 2:**
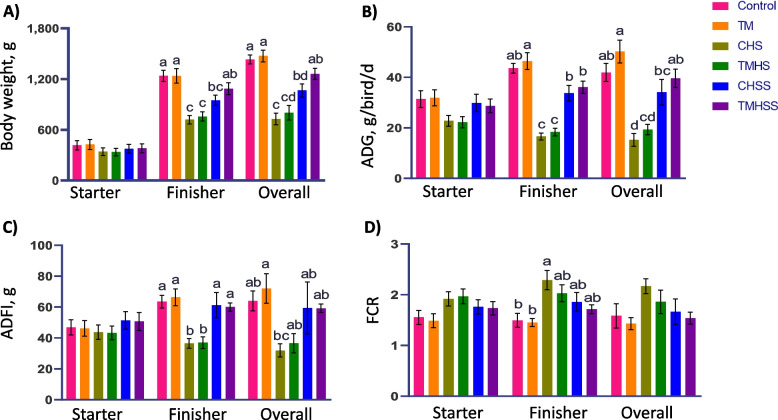
Effects of TM and baicalein supplementation on broiler’s growth performance. **A**) Body weight; **B**) ADG; **C**) ADFI; **D**) FCR. Data showed as mean ± SEM. The effects of treatments were significantly different at *P* < 0.05 for body weight, ADG, ADFI, and FCR. ^a-d^Different letters indicate a significant difference among the treatment groups

The overall mortality rate during the experimental period was 4% in the Control and TM group, followed by 16% in CHS, 13% in TMHS, 12% in CHSS, and 10% in TMHSS. These dead birds were excluded from the growth performance data. Overall body weight on d 35 was significantly higher (*P* < 0.05) in the Control and TM groups compared to the CHS, TMHS, and CHSS groups (Fig. 2). Simultaneously, the baicalein supplementation increased the body weight of the TMHSS group. ADG was significantly lower (*P* < 0.05) in CHS and TMHS groups compared to the Control group on d 35. Also, the ADG was numerically higher in the TM group than in the Control group. Similarly, the supplement helped to improve the ADG in the TMHS group under heat stress conditions compared to the CHSS group. Overall (d 1–35) ADFI significantly decreased (*P* < 0.05) in the CHS group compared to the TM group. Baicalein numerically improved ADFI in both CHSS and TMHS groups despite heat stress. FCR was significantly higher (*P* < 0.05) in the CHS group in the finisher phase compared to the Control and TM groups. However, overall, there was no significant difference among the treatment groups. Nevertheless, the FCR numerical value in the TM group was the lowest among all groups. Also, the TMHS group improved FCR with the baicalein supplement, which was lower than the Control group.

### Intestinal gene expression

**Fig. 3:**
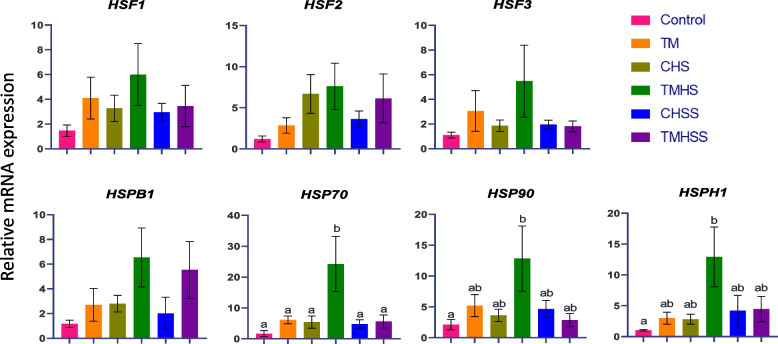
Effects of TM and baicalein supplementation on the mRNA expression of heat shock genes. Data showed as mean ± SEM. ^a,b^Different letters indicate a significant difference among the treatment groups

The expression pattern of the heat shock protein-related genes (*HSF1, HSF2, HSF3, HSPB1, HSP70, HSP90*, and *HSPH1*) among the treatment is summarized in Fig. 3. The mRNA expression of *HSF1, HSF2,* and *HSF3* showed an improvement in the TMHS group. In the case of *HSPB1*, the TMHS and TMHSS groups' relative mRNA expression was higher than the Control group. The expression of *HSP70* was significantly increased (*P* < 0.05) in the TMHS group compared to all the treatment groups. *HSP90* and *HSPH1* expression were significantly increased (*P* < 0.05) in the TMHS group compared to the Control group.

**Fig. 4:**
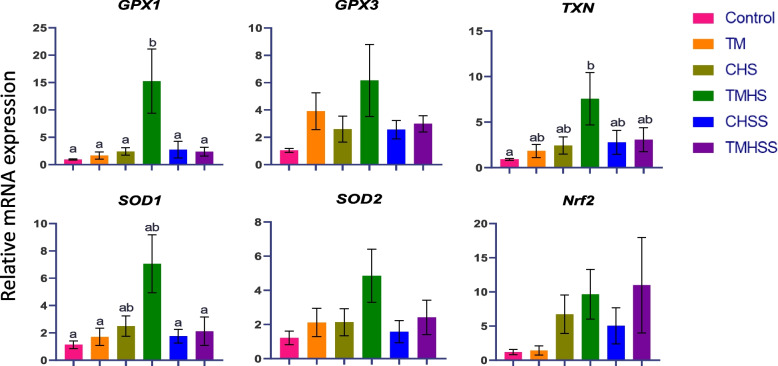
Effects of TM and baicalein supplementation on the mRNA expression of antioxidant genes. Data showed as mean ± SEM. ^a,b^Different letters indicate a significant difference among the treatment groups

The expression of antioxidant-related genes (*GPX1, GPX3, TXN, SOD1, SOD2,* and *Nrf2*) is depicted in Fig. 4. The mRNA expression of *GPX1* and *TXN* was significantly higher (*P* < 0.05) in the TMHS group compared to the Control group*. GPX3* had a higher mRNA expression in TM and TMHS groups. *SOD1* and *SOD2* mRNA expression improved in the TMHS group compared to the other treatments. The expression of the *Nrf2* was enhanced in the Baicalein supplementation group TMHSS.

### Cecal volatile fatty acids

**Fig. 5:**
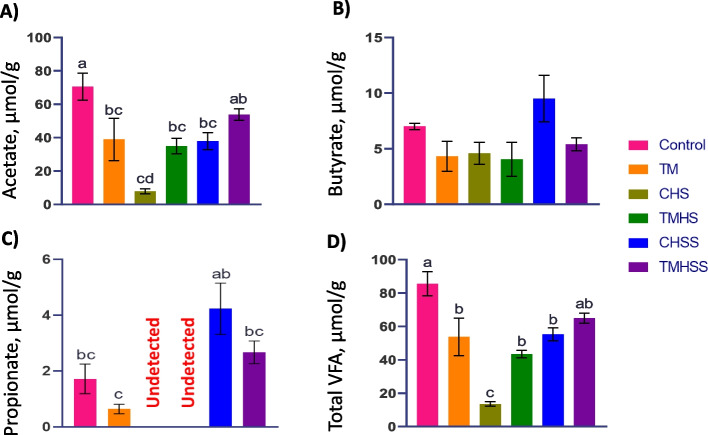
Effects of TM and baicalein supplementation on the important volatile fatty acids in the cecal digesta. **A**) Acetate; **B**) Butyrate; **C**) Propionate; **D**) Total VFA. ^a–d^Data showed as mean ± SEM. Different letters indicate a significant difference among the treatment groups

The acetate concentration was significantly lower (*P* < 0.05) in the TM, CHS, CHSS, and TMHS groups compared to the Control group. At the same time, dietary baicalein supplementation improved acetate concentration in the TMHSS group (Fig. 5). There was no significant change (*P* > 0.05) in butyrate concentration among the treatment groups. Propionate concentration was minimal (undetected) in the CHS and TMHS groups. Overall, the TM, CHS, TMHS, and CHSS groups have significantly lower (*P* < 0.05) VFA concentrations than the Control group. However, the baicalein supplementation improved the total VFA concentration of the TMHSS group under the heat stress condition.

### Alpha and Beta diversity of cecal microbiota

**Fig. 6:**
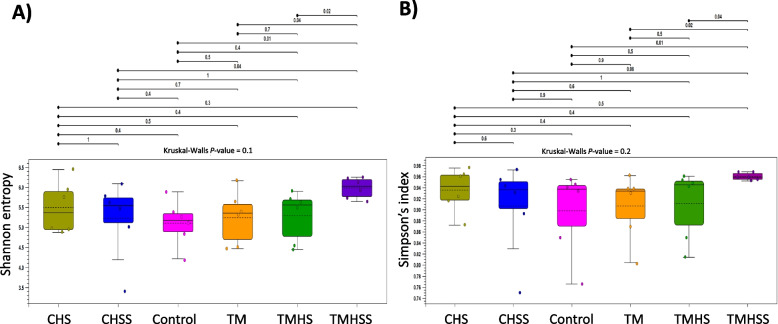
Effects of TM and baicalein supplementation on microbial alpha diversity. **A**) Shannon entropy; **B**) Simpson’s index. The effects of treatments were significantly different at *P* < 0.05 among the treatment groups

Alpha diversity measures the diversity of microbiota within the treatments. This study used Shanon entropy and Simpson’s index to measure the Alpha diversity (Fig. 6). The Shannon entropy quantifies uncertainty regarding the identity of sample species. It evaluates species richness. Simpson’s index represents a probability, particularly the probability that two randomly selected individuals from the sample will belong to different species. It assesses relative abundance. In this study, Shannon entropy showed a significant difference (*P* < 0.05) in the TMHSS group compared to the Control, TM, TMHS, and CHSS groups. Simpson's index was significantly different (*P* < 0.05) in the TMHSS group compared to the Control, TM, and TMHS groups.

**Fig. 7:**
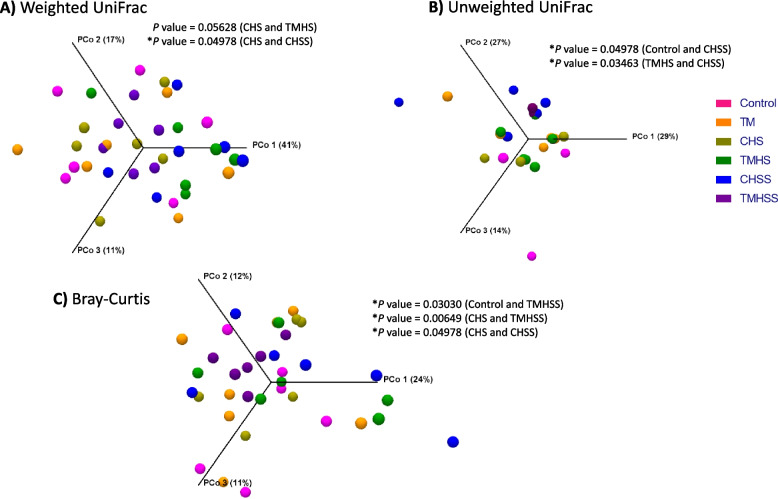
Effects of TM and baicalein supplementation on microbial beta diversity. **A**) Weighted UniFrac; **B**) Unweighted UniFrac; **C**) Bray–Curtis. The effects of treatments were significantly different at *P* < 0.05 among the treatment groups

Beta diversity measures the difference in microbial composition between different treatment groups. This study measured Beta diversity using weighted UniFrac, unweighted UniFrac, and Bray–Curtis (Fig. 7). The weighted UniFrac shows a significant difference in the CHS group compared to the CHSS group (*P* = 0.04978). The unweighted UniFrac depicts a significant difference in the CHSS group compared to Control (*P* = 0.04978) and TMHS group (*P* = 0.03463). The Bray–Curtis revealed a significant difference in the TMHSS group compared to the Control (*P* = 0.03030) and CHS group (*P* = 0.00649). Furthermore, it also showed a significant difference between the CHS and CHSS group (*P* = 0.04978).

### Cecal microbiota profile

**Fig. 8:**
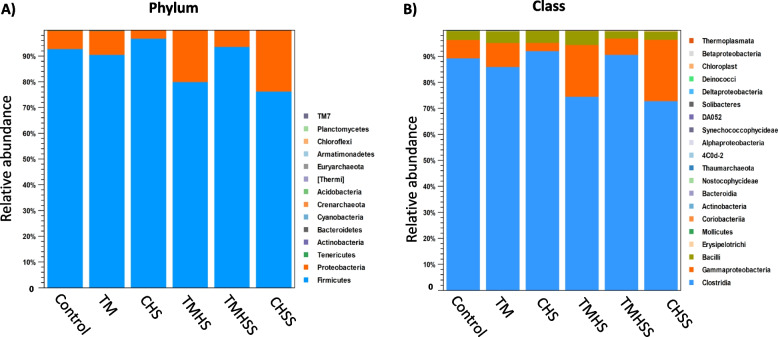
Effects of TM and baicalein supplementation on the average relative abundance of the microbiota at the phylum (**A**) and class (**B**) level

This study explored the cecal microbial profiling at phylum, class, order, family, and genus levels. The cecal microbiota diversity at the phylum level among treatment groups is presented after removing OTUs with low abundance (Fig. 8A). Firmicutes and Proteobacteria were the two most prevalent phyla among the treatments; in the Control (93% and 7%, respectively), TM (90% and 10%, respectively), CHS (98% and 2%, respectively), TMHS (80% and 20%, respectively), TMHSS (96% and 4%, respectively), and CHSS (76% and 24%, respectively).

At the class level (Fig. 8B), the microbiota was dominated by Clostridia and Gammaproteobacteria. They were present in Control (89% and 7%, respectively), TM (86% and 8%, respectively), CHS (93% and 2%, respectively), TMHS (74% and 20%, respectively), TMHSS (90% and 8%, respectively), and CHSS (73% and 22%, respectively).

**Fig. 9:**
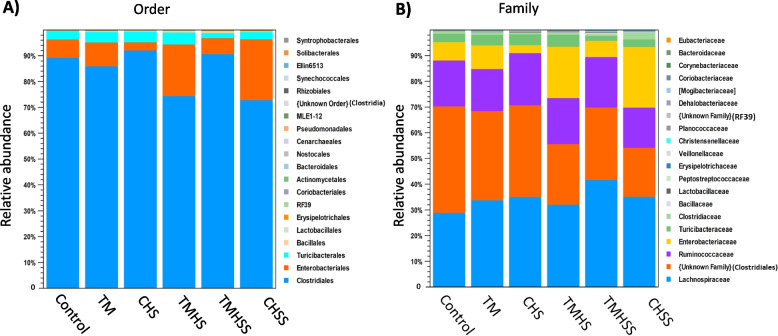
Effects of TM and baicalein supplementation on the average relative abundance of the microbiota at the order (**A**) and family (**B**) levels

At order level (Fig. 9A), the microbial composition was enriched by Clostridiales, Enterobacteriales, and Turicibacteriales, which included in the Control (89%, 7%, and 3%, respectively), TM (86%, 9%, and 4%, respectively), CHS (93%, 2% and 4%, respectively), TMHS (74%, 19% and 5%, respectively), TMHSS (92%, 6% and 1%, respectively), and CHSS (73%, 22% and 4%, respectively).

Lachnospiraceae, Unknown family (Clostridiales), Ruminococcaceae, and Enterobacteriaceae were the predominant bacterial composition at the family level (Fig. 9B). Their abundance includes in Control (29%, 41%, 18%, and 6%, respectively), TM (34%, 36%, 16% and 7%, respectively), CHS (35%, 41%, 19%, and 2%, respectively), TMHS (32%, 24%, 16% and 22%, respectively), TMHSS (44%, 26%, 20%, and 5%, respectively) and CHSS (38%, 19%, 14% and 24%, respectively).

**Fig. 10:**
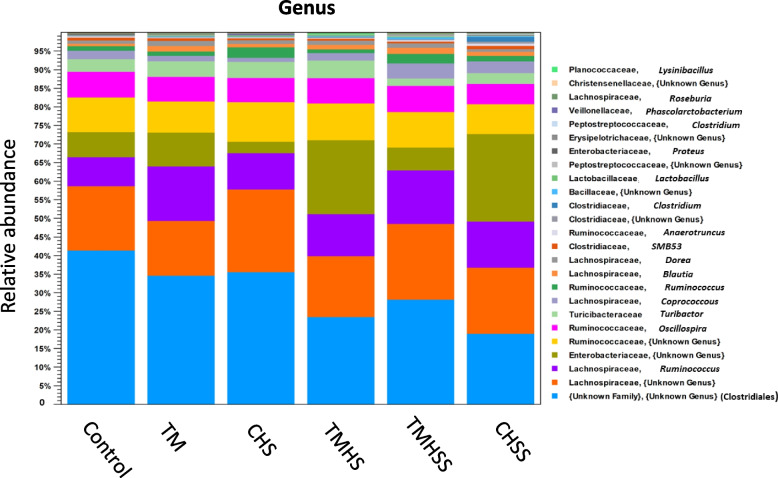
Effects of TM and baicalein supplementation on the average relative abundance of the microbiota at the genus levels

At the genus level (Fig. 10), the microbial composition was enriched by the Unknown genus (Clostridiales), Unknown genus (Lechnospiraceae), *Ruminococcus*, Unknown genus (Enterobacteriaceae), Unknown genus (Ruminococcaceae), and *Oscillospira*, which includes in the Control (41%, 18%, 6%, 7%, and 8%, respectively), TM (34%, 14%, 15%, 8%, and 7%, respectively), CHS (35%, 25%, 8%, 3%, and 11%, respectively), TMHS (24%, 16%, 10%, 22%, and 7%, respectively), TMHSS (28%, 20%, 12%, 5%, and 10%, respectively) and CHSS (20%, 18%, 12%, 25%, and 6%, respectively).

### Specific bacterial abundance in cecal microbiota

**Fig. 11:**
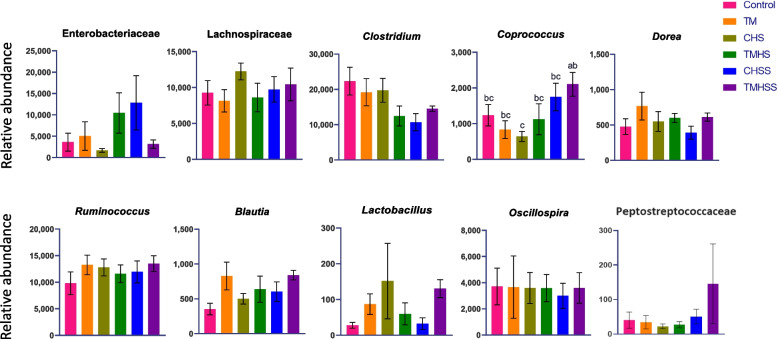
Effects of TM and baicalein supplementation on significantly abundance microbiota at the family and genus level.

Specific bacterial abundance at the family and genus levels is presented in Fig. 11. At the family level, Enterobacteriaceae and Lechnospiraceae abundance were not significantly different among treatments (*P* < 0.05). However, Peptostreptococcaceae had improved abundance in the Baicalein supplement group TMHSS. *Ruminococcus, Blautia, Clostridium, Dorea, Lactobacillus*, and *Oscillospira* bacterial opulence were not significantly changed at the genus level. However, *Coprococcus* was significantly increased in the TMHSS group compared to the CHS group.

## Discussion

Chickens exposed to high ambient temperatures reduce feed intake to maintain body temperature [16]. It is reported that broiler chickens had a 3.4% decrease in feed intake per degree increase in temperature between 21 and 34 °C in Arbor Acres broilers and a 1.7% decrease in Beijing-You chickens (a Chinese slow-growing breed) [17]. This study found that cyclic heat stress significantly decreased the final body weight and ADG in CHS and TMHS groups on d 35. In contrast, the baicalein supplementation in the TMHSS group improved those parameters even though they were in the heat stress condition. There was no significant change in ADFI and FCR among the treatment groups. However, TM, TMHS, and TMHSS groups had better FCR and ADFI in the heat-stressed environment, along with improved antioxidant and heat shock protein-related gene expression. Besides that, TMHS and TMHSS groups had improved total VFA concentration and enhanced relative beneficial bacterial abundance in the cecum. All these phenomena imply that pre-hatch TM had a long-term beneficial effect on growth parameters and gut health with or without HS conditions. In addition, 250 mg/kg baicalein supplementation with TM (TMHSS group) had better body weight gain and feed utilization in heat-stressed birds.

To comprehend the potential mechanism of TM and dietary baicalein supplements, we analyzed important genes associated with antioxidant and heat stress in poultry from ileum samples. The ileum, the final section of the small intestine, ends at the ileo-ceco-colic junction. In fast-growing broiler chickens, the ileum is crucial in digesting and absorbing nutrients, including carbohydrates [18]. The gastrointestinal tract is particularly sensitive to stress, including heat stress. The functionality of the intestinal tract is essential for poultry production because it has far-reaching effects on the birds' overall welfare and productivity [19]. The heat stress response begins with the phosphorylation and trimerisation of heat shock factors (HSF). These trimers then translocate to the nucleus and attach with the heat shock elements in the promoter region of heat shock protein (*HSP*) genes, thereby regulating *HSP* transcription. HSPs play a crucial role in repairing and preserving the internal environment by promoting the degradation of misfolded proteins and aiding in protein refolding. *HSF3* is the master regulator of *HSP* expression in avian species, as in *HSF1* in mammals. However, HSF1 and HSF3 demonstrate DNA-binding capability and are translocated to the nucleus in response to heat shock, whereas only a minor amount of *HSF2* does [20]. HSF2 is a key regulator of proteostasis and helps *HSF3* to express against febrile-range thermal exposure [21]. This study showed improved gene expression in *HSF1, HSF2*, and *HSF3* in the TMHS group.

However, the expression pattern of these genes was numerically higher in both TMHS and TMHSS groups compared to the Control group. This result positively correlates with *HSPB1 (HSP27), HSP70, HSP90,* and *HSPH1 (HSP110)* expression in this study. As HSFs were improved in the TMHS group, they regulated the expression of HSPs under high ambient temperatures. Due to this reason, the *HSP70, HSP90,* and *HSPH1* expressions were significantly upregulated (*P* < 0.05) in the TMHS group than in the Control group. The representation trend was higher in the TMHS and TMHSS groups in the case of the *HSPB1*. The upregulation of HSPs, especially *HSP70,* is considered to have an inhibition mechanism against the expression of pro-inflammatory cytokines [22]. HSP70 can also stimulate glutathione peroxidase (*GPX*) and reductase (*GR*) [23]. HSP90 is linked to proteins in later stages of growth and modifies their morphology [24]. HSP110 can chaperone antigens for presentation to T lymphocytes and intensify signals that trigger inflammation in the extracellular environment, indicating that it may have natural immunostimulatory effects during tissue stress or injury [25]. *HSP27* is linked to safeguarding against heat-induced cardiomyocyte damage and apoptosis [26]. Therefore, after analyzing the cascade of events induced by HSPs, it is obvious that HSP-mediated thermotolerance is essential for the survival and adaptation of chicken cells under thermal stress conditions. Our results suggest that TM during ED 12 to 18 alters the gene expression of important HSPs. This indicates an enhanced thermotolerance acquisition in chickens subjected to TM under the heat stress condition, particularly in the finisher phase.

The reactive oxygen species (ROS) and reactive nitrogen species (RNS) ratio are balanced in normal physiological conditions. ROS/RNS are crucial signaling molecules, the antioxidant defense system meticulously regulates their concentration and numerous transcription factors. Birds that experience heat stress have an excess ROS/RNS concentration in the body. Activation of vitagenes by transcription factors starts via *Nrf2*, synthesizing a wider variety of protective molecules that can combat a spike in ROS/RNS production. Then it triggers the three main antioxidant enzymes, superoxide dismutase (SOD), GPX, and catalase, responsible for radical detoxification at the beginning of their formation [27]. To understand how TM and flavonoids supplement influence antioxidant activity, we analyzed some important antioxidant-related genes and their complex procedure of the cellular defense network. *Nrf2* was not significantly different among treatments in this study. However, the expression was numerically higher in TMHS and TMHSS groups than in other groups, which may pave the way to express other antioxidant-related genes under the heat stress condition. As a result, *GPX1* is significantly upregulated (*P* < 0.05), and *GPX3* had an improved expression in the TMHS group among the treatment groups. In avian species, *GPX1* and *GPX3* are the selenium-dependent glutathione forms involved in the primary and secondary levels of the antioxidant network. *GPX1* is primarily found in the cytoplasm and mitochondria, whereas *GPX3* is prevalent in plasma. GPXs catalyze glutathione's reduction of hydroperoxides and H_2_O_2_ [28]. It has been reported that increased synthesis of *SOD1* and *SOD2* in response to stress is an adaptive mechanism for reducing ROS formation, preventing oxidative stress, and sustaining adaptive homeostasis. *SODs* catalyze the transformation of superoxide to hydrogen peroxide [29]. In this study*, SOD1* and *SOD2* expression in the ileum was increased in both TMHS and TMHSS groups which strongly correlates with the increasing trend of *Nrf2*, *GPX1,* and *GPX3* genes. These genes are related to the first and second lines of defense mechanisms against heat stress. It means that thermal acquisition is more active in the thermally conditioned embryo at the early stage of development. This evidence can be supported by looking at the expression pattern of the *TXN*. In this study, *TXN* was significantly upregulated (*P* < 0.05) in the TMHS group. The *TXN* and *GPX* systems can be each other's fallback in mammalian cells. *TXN* is a crucial antioxidant system in the body's protection against oxidative stress because its disulfide reductase activity regulates the protein dithiol/disulfide equilibrium [30]. So, it is evident that embryonic TM and flavonoids in baicalein supplements may play a key role in upregulating the antioxidant-related genes in heat-stressed chickens by minimizing lipid peroxidation and ROS/RNS at the cellular level.

The VFA produced during the cecal fermentation are crucial biomarkers for assessing the intestinal health of poultry. Acetate, propionate, butyrate, lactate, valerate, and isovalerate are the main VFAs [31]. Among them, acetate, propionate, and butyrate are the most important. VFAs can increase barrier function, regulate microorganisms, boost immune function, and prevent inflammation of the gastrointestinal tract in poultry. Acetate, a predominant VFA, is associated with the muscle glycolytic pathway and can decrease luminal pH, thereby preventing the colonization of pathogenic microorganisms [32] and significantly influencing colibacillosis [33]. This study showed a significant decrease of acetate in TM, CHS, TMHS, and CHSS groups compared to the Control group. However, acetate production was similar in the TMHSS group even though they were exposed to heat stress which can be explained by their improved antioxidant gene expression shown in the study. It may help to protect intestinal immunity by increasing beneficial bacterial abundance in the TMHSS group. Butyrate not only supplies energy to colonocytes, but it is also a cellular mediator that regulates multiple functions of gut cells and beyond, such as gene expression, cell division, gut tissue growth and development, immune regulation, oxidative stress reduction, and diarrhea control [34]. However, this study found no significant difference in butyrate production among treatments. Propionate is associated with gluconeogenesis and regulation of fat metabolism in the liver and can inhibit fat deposition in the abdomen [35]. Propionate production was minimal (undetected, insufficient data to run the statistics) in both CHS and TMHS groups. It can be an important reason for reduced final body weight in these groups in the study. TM group had a significantly lower propionate production than the CHSS group. Regarding total VFA production, the CHS group produced significantly lower VFA than the other treatment groups. This result can be attributed to the beneficial effect of embryonic TM and post-hatch baicalein supplements. In addition, these VFA results strongly correlated with our finding of enhanced growth performance, antioxidant gene expression, and relative bacterial enrichment.

The gut contains trillions of commensal microbes living in symbiosis with the host, constituting an intricate microbial ecosystem. Interactions between the host and the gastrointestinal microbiome are essential for poultry growth, health, and nutrition. The baicalein significantly improved the variety and diversity of the cecal microflora in the TMHSS birds, which may be credited to the flavonoids in the dietary supplement. In this study, TMHS and TMHSS groups showed a significant change in weighted UniFrac, unweighted UniFrac, and Bray–Curtis measurements in beta diversity. Previous studies [15, 36], support this remarkable shift in microbial diversity in heat stress conditions and indicate that the relative abundance of bacteria substantially differed between treatments. Flavonoids may help to stabilize the free radicals by binding with them. Thus, keeping the gut integrity intact prevents the dysbiosis of the cecal microbiota.

The gastrointestinal tract has evolved to perform two seemingly special functions: absorbing nutrients and defense against pathogens as a vital organ of the mucosal immune system of the host. It is well known that a healthy and beneficial microbial community plays a crucial role in sustaining normal physiological homeostasis, regulating the host immune system, and influencing organ growth and host metabolism [37]. Our study reveals that Firmicutes, Proteobacteria, Tenericutes, and Actinobacteria are commonly present in the phylum level where Firmicutes are the dominant population. In phylum, class, order, family, and genus levels, the TMHSS and CHSS groups had the most balanced bacterial population than the Control group. The reason might be the baicalein supplement, which helps express the antioxidant genes at a certain level, suppressing the negative immune response and preventing dysbiosis. Thus, it keeps the intestinal integrity intact in a heat-stressed environment.

To further investigate the gut microbial community in the birds, we delve deeper and analyze significant microbiota abundance at the family and genus levels. At the family level, Enterobacteriaceae, Lechnospiraceae, and Peptostreptococcaceae were dominant. However, those bacteria were not significantly different among the treatment groups. Enterobacteriaceae contains molecules that amplify the inflammatory response directly. The bacterial surface presents molecules called Microbe-associated molecular patterns (MAMPs). These molecules interact with immune cell receptors to induce inflammation [38]. Lachnospiraceae are extremely prevalent in poultry and are especially efficient at degrading cellulose and other non-digestible polysaccharides [39]. The Peptostreptococcaceae population was increased in the TMHSS group compared to the other treatments. Peptostreptococcaceae is a family of Gram-positive bacteria belonging to the Clostridia class. They are the typical commensal of the gut. Its proportion is greater in the gut microbiota of healthy animals than in those with dysbiosis of the intestinal microbiota, indicating that Peptostreptococcaceae contributes to maintaining gut homeostasis [40]. It is also involved in the biosynthesis pathway that produces glutamate to butyrate. This taxonomic group can produce VFAs from amino acids, even though Peptostreptococcaceae is not a typical butyrate producer. The pathway from amino acids to butyrate may enhance gut VFA production from a limited carbon field source [41].

At the genus level, the *Ruminococcus*, *Blautia*, *Clostridium*, *Dorea*, *Lactobacillus*, and *Oscillospira* population were not significantly different among the treatment group. TMHS and TMHSS groups had an improved *Ruminococcus* concentration in the cecum. *Ruminococcus* are a significant butyrate-producing bacteria. It produces butyrate through the fermentation of carbohydrates by converting two acetyl-CoA molecules into crotonyl-CoA [42]. *Blautia* is a gut microbial genus that produces butyric acid and acetic acid, which reduce abdominal fat accumulation (inversely related) by modulating G-protein coupled receptors (GPR) 41 and 43 [43]. *Blautia* population was higher in the TM and TMHSS birds. We also noticed less abdominal fat in these groups while sampling. It is also known for its antibacterial function against specific microbiota. It contributes to mitigating inflammatory diseases and metabolic disorders [44]. *Dorea* has pro- or anti-inflammatory properties, relying on gut flora and/or nutrients. As *Blautia* utilizes gases produced by *Dorea*, the increased abundance of *Dorea* in multiple sclerosis patients may facilitate the development of *Blautia* in humans [45]. Clostridium is a common inhibitor in the gut. Significant human and animal pathogens, such as tetanus and botulism, are included in this genus [46]. We found less Clostridium abundance in the TMHS, CHSS, and TMHSS groups. That may be because of the anti-inflammatory activity of the baicalein supplement, and TM birds may cope with the HS as they grow older (as found at d 35).

*Oscillospira* concentration was not significantly different among the treatments. A study has demonstrated a significant positive correlation between *Oscillospira* and low body fat, leanness, and human health, as well as a strong connection between this organism and several diseases, particularly inflammatory diseases [47]. Lastly, *Coprococcus* concentration in the cecal microbiota was significantly higher in the TMHSS group compared to the CHS group and had more abundance among the other groups. It is associated with maintaining intestinal microbial homeostasis, producing essential VFAs and vitamin B-complexes, and host immune function [48]. It is worth noting that our supplement, baicalein, is a bioactive compound that is beneficial for growth and promoting the gut microbiome. In this study, we found that early TM and post-hatch baicalein supplementation favor enhancing beneficial bacteria in the gut, which helps maintain normal immune status and thus promotes the growth performance of the broiler chicken. However, further investigation is needed to understand its effects on the gut microbial community as their function is very complex.

## Conclusion

The embryonic TM and post-hatch baicalein supplementation can improve the body weight, ADG, ADFI, FCR, and cecal VFA production in broiler chickens. TM alone can significantly upregulate the expression of important heat shock and antioxidant-related genes in heat-stressed birds. Combining TM with baicalein supplementation improves the relative bacterial abundance of beneficial bacteria in the chicken gut. Therefore, pondering the benefits of embryonic TM and post-hatch baicalein supplements can be considered a viable strategy to mitigate the adverse effect of heat stress in broiler chickens.

### Supplementary Information

**Additional file 1: Table S1.** Primers used to quantify the expression of target genes by qPCR.

## Data Availability

The metagenomics sequence data used in this study have been submitted to the NCBI database (accession no: PRJNA979953).

## Abbreviations

ADG: Average daily gain
ADFI: Average daily feed intake
ANOVA: Analysis of variance
Ct: Cycle threshold
cDNA: Complementary deoxyribonucleic acid
ED: Embryonic day
FCR: Feed conversion ratio
GAPDH: Glyceraldehyde 3-phosphate dehydrogenase
GPR: G-protein coupled receptors
GPX1: Glutathione peroxidase 1
GPX3: Glutathione peroxidase 3
GR: Glutathione reductase
HS: Heat stress
HSF1: Heat shock factor 1
HSF2: Heat shock factor 2
HSF3: Heat shock factor 3
HSPB1: Heat shock protein B1
HSP70: Heat shock protein 70
HSP90: Heat shock protein 90
HSPH1: Heat shock protein H1
MAMP: Microbe-associated molecular patterns
MSA: Multiple sequence alignment
Nrf2: Nuclear factor-erythroid factor 2-related factor 2
OUT: Operational taxonomical unit
PCA: Principal coordinate analysis
PERMANOVA: Permutational multivariate analysis of variance
RH: Relative humidity
RNS: Reactive nitrogen species
ROS: Reactive oxygen species
SOD1: Superoxide dismutase 1
SOD2: Superoxide dismutase 2
TBP: TATA-box binding protein
TM: Reactive nitrogen species
TXN: Thioredoxin
VFA: Volatile fatty acid
