# Supplementary material for: Pre-hatch thermal manipulation of embryos and post-hatch baicalein supplementation mitigated heat stress in broiler chickens
Source: J Anim Sci Biotechnol. 2024 Jan 22;15:8. doi: 10.1186/s40104-023-00966-6 (PMC10802028; doi:10.1186/s40104-023-00966-6)
Supplement: Supplementary file 1 — Additional file 1: Table S1. Primers used to quantify the expression of target genes by qPCR. [file 40104_2023_966_MOESM1_ESM.docx]

**Supplement Table 1.** Primers used to quantify the expression of target genes by qPCR.

| **Gene** | **Accession no.** | **Primer sequence** | **Amplicon length** |
| --- | --- | --- | --- |
|  |  |  |  |
| *TBP* | XM_025148547.3 | F: TAGCCCGATGATGCCGTAT | 147 |
|  |  | R:GTTCCCTGTGTCGCTTGC |  |
| *GPX1* | NM_001277853.2 | F: AATTCGGGCACCAGGAGAA | 101 |
|  |  | R: CTCGAACATGGTGAAGTTGG |  |
| *GPX3* | NM_001163232.2 | F:GAGGGAGAAGGTGAAATGCT | 192 |
|  |  | R:CCCAGCTCATTTTGTAGTGC |  |
| *HSF1* | NM_001305256.1 | F:AAGGAGGTGCTCCCAAAGTA | 221 |
|  |  | R:TTCTTTATGCTGGACACGCTG |  |
| *HSF2* | NM_001167764.2 | F:TCT TTT TAC AAG CTC CGT GC | 70 |
|  |  | R:TCC CTT TGT CTC CAT TTT GGT |  |
| *HSF3* | NM_001305041.1 | F:TTCAGCGATGTGTTTAACCCT | 244 |
|  |  | R:GGAGGTCTTTTGGATCCTCT |  |
| *HSP70* | NM­_001006685.1 | F:TCTCATCAAGCGTAACACCAC | 104 |
|  |  | R:TCTCACCTTCATACACCTGGAC |  |
| *HSP90* | NM_001109785.1 | F:GATAACGGTGAACCTTTGGG | 120 |
|  |  | R:GGGTAGCCAATGAACTGAGA |  |
| *SOD1* | NM_205064.1 | F: CAACACAAATGGGTGTACCA | 119 |
|  |  | R: CTCCCTTTGCAGTCACATTG |  |
| *SOD2* | NM_204211.1 | F: CCTTCGCAAACTTCAAGGAG | 160 |
|  |  | R: AGCAATGGAATGAGACCTGT |  |
| *TXN* | NM_205453.1 | F: GGCAATCTGGCTGATTTTGA | 79 |
|  |  | R: ACCATGTGGCAGAGAAATCA |  |
| *Nrf2* | NM_001396038.1 | F: CCCTGCCCTTAGAGATTAGAC | 248 |
|  |  | R:CAAGTTCATGTCCTTTTCTCTGC |  |
| *HSPH1* | NM_001159698.2 | F: AAACTGATGGTCAGCAAACG | 239 |
|  |  | R: TTGCATGATCATCTTACCCTCT |  |
| *HSPB1* | NM_205290.2 | F: GAGATCACCGGCAAACACG | 184 |
|  |  | R: TGATCTCGGATGACTGGATG |  |
